# Supplementary material for: Integrating genomic structural equation modeling and experimental validation to unravel the genetic basis of male genital lichen sclerosus
Source: Front Immunol. 2026 Jul 16;17:1857945. doi: 10.3389/fimmu.2026.1857945 (PMC13435250; doi:10.3389/fimmu.2026.1857945)
Supplement: Supplementary file 6 [file SupplementaryFile5.zip › result/fuma_PSYCH_factor_sCCA1_focus.chr6.chr6.103983395.106056733.pop1.pdf]

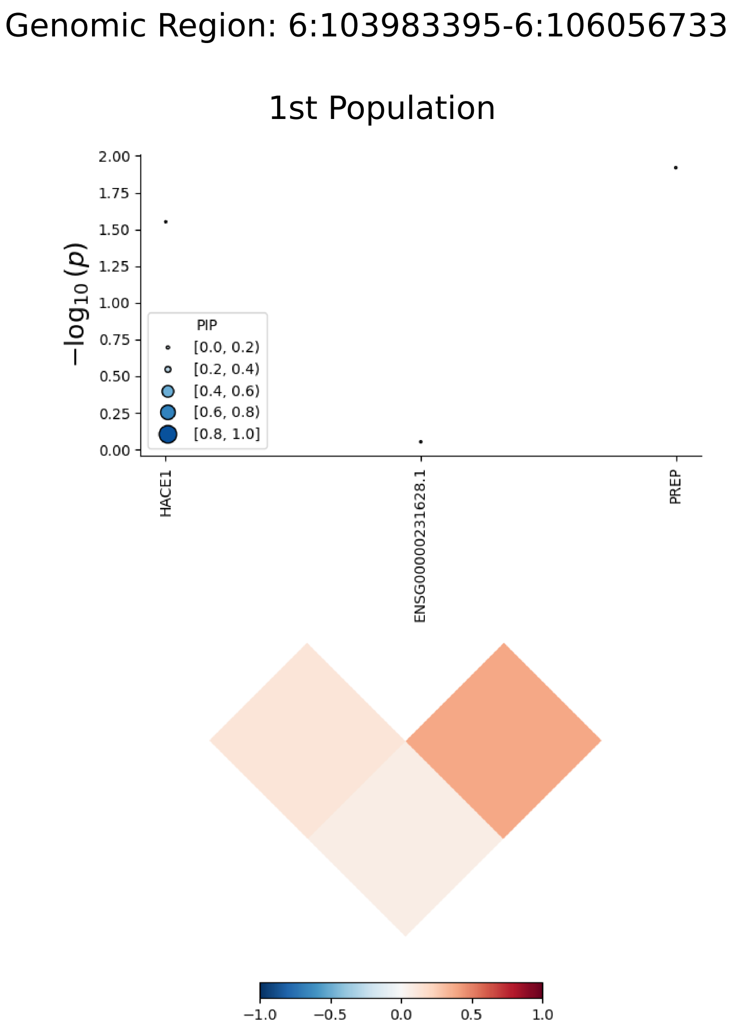
Genomic Region: 6:103983395-6:106056733
1st Population
2.00
1.75
1.50
1.25
1.00
0.75
0.50
0.25
0.00
$-\log_{10}(p)$
PIP
[0.0, 0.2)
[0.2, 0.4)
[0.4, 0.6)
[0.6, 0.8)
[0.8, 1.0]
HACE1
ENSG00000231628.1
PREP
−1.0
−0.5
0.0
0.5
1.0
